# Comparative Analysis of HSF Genes From *Secale cereale* and its Triticeae Relatives Reveal Ancient and Recent Gene Expansions

**DOI:** 10.3389/fgene.2021.801218

**Published:** 2021-11-23

**Authors:** Xiao-Tong Li, Xing-Yu Feng, Zhen Zeng, Yang Liu, Zhu-Qing Shao

**Affiliations:** School of Life Sciences, Nanjing University, Nanjing, China

**Keywords:** *S. cereale*, HSF gene, stress tolerance, evolution, functional diversity

## Abstract

Plants have evolved sophisticated systems to cope with the environmental stresses, with the heat shock factor (HSF) family proteins composing an integral part of the transcriptional regulation system. Understanding the evolutionary history and functional diversity of HSFs will facilitate improving tolerance of crops to adverse environmental conditions. In this study, genome-wide analysis of *Secale cereale* identified 31 HSF genes. The total number of HSF genes in *S. cereale* is larger than that in barley and the three subgenomes of wheat, suggesting it is a valuable resource for mining functional HSFs. Chromosome analysis revealed an uneven distribution of HSF genes among the 7 *S. cereale* chromosomes, with no HSF gene was detected on chromosome 4. Further interspecies synteny analysis revealed that chromosome reorganization during species-speciation may lead to the escape of HSF genes from the *S. cereale* chromosome 4. Phylogenetic analysis revealed that *S. cereale* experienced more HSF gene duplications than barley and the three wheat subgenomes. Expression analysis demonstrated that *S. cereale* HSF genes showed diverse expression patterns across plant developmental stages and upon drought and freezing treatment, suggesting functional diversity of the gene family. Notably, we detected distinct expression patterns for a recently duplicated HSF gene pair, indicating functional divergence may have occurred between the two genes. The study presents the genome organization, evolutionary features and expression patterns of the *S. cereale* HSF genes. These results provide new insights into the evolution of HSF genes in Triticeae and may serve as a resource for Triticeae molecular breeding.

## Introduction

Plants are consistently affected by biotic and abiotic stresses in the environment during their whole lifespan, including drought, salt, heat, cold and pathogens’ infection. In the long-term evolution, plants have evolved sophisticated systems and regulatory networks to avoid or attenuate the deleterious effects of these stresses (Peck and Mittler, 2020). Many gene families have been reported for their distinct roles in responding to external environmental stresses, such as the nucleotide-binding leucine-rich-repeat (NLR) disease resistance gene family (Saur et al., 2021); and the cold-induced C-repeat binding factor (CBF) gene family (Zhou et al., 2011). There are also some gene families that can respond to multiple external stresses by their different family members (Javed et al., 2020; Wani et al., 2021). Among them, plant heat shock factor (HSF) family proteins compose an integral part of the transcriptional regulation system for plants against external environmental stresses, by modulating the expression of different sets of plant genes in responding to heat, cold, salt stresses and the infection of pathogens (Andrasi et al., 2021).

HSF is a conserved gene family that widely spreads in eukaryotes and prokaryotes. Proteins encoded by this family were initially identified as transcription factors that regulate the expression of HSPs, whose functions as molecular chaperones to maintain protein homeostasis in cells (Boston et al., 1996). However, increasing studies in plants have revealed that HSFs are important components of the complex signaling systems that control responses not only to high temperatures but also to a number of abiotic stresses such as cold, drought, hypoxic conditions, soil salinity, and to pathogen threats (Andrasi et al., 2021). Compared to the single copy HSF gene in yeast and four HSF genes in human genome, plant genomes have expanded the HSF genes to several dozens (Wang et al., 2018). For example, 22 and 25 HSF genes have been identified from the dicot plant *Arabidopsis thaliana* and monocot species *Oryza sativa*, respectively (Guo et al., 2008).

The plant HSF family proteins have several characteristic domains that are essential for their functions, including a N-terminal DNA-binding domain (DBD) that recognizes heat shock elements in the promoter region of target genes, a following oligomerization domain (OD or HR-A/B motif) that is responsible for protein–protein interactions and trimerization during transcriptional activation (Scharf et al., 2012). Based on the length of the linker between the DBD and OD domains and the number of amino acid residues inserted into the HR-A/B regions, plant HSFs are classified into three subgroups: HSFA, HSFB and HSFC (Scharf et al., 2012). The subgroup A HSFs have additional nuclear localization signal (NLS) and nuclear export signal (NES) sequences, and a C-terminal aromatic and hydrophobic amino acid motif (AHA), which is needed for its transcriptional activation activity (Andrasi et al., 2021). The subgroup B HSFs contain a C-terminus tetrapeptide (LFGV) that functions as a repressor domain (RD) (Scharf et al., 2012; Andrasi et al., 2021).

Due to the functional importance, genome-wide identification and functional exploration of HSF genes have been carried out in model plants and many crops (Wang et al., 2018; Andrasi et al., 2021). Some HSF members have been used to enhance plant tolerance to different stresses and molecular breeding. For example, overexpression of *AtHSFA2* in *A. thaliana* increased the motolerance, salt/osmotic stress tolerance, and enhanced callus growth of the plant (Charng et al., 2007; Ogawa et al., 2007; Nishizawa et al., 2006). Overexpression of *GmHSFA1* in soybean and *SlHSFA1* in tomato enhanced thermotolerance of the transgenic plants (Mishra et al., 2002; Zhu et al., 2006).

Triticeae crops, including wheat (*Triticum aestivum*), barley (*Hordeum vulgare*) and rye (*Secale cereale*), are important grain crops, which are frequently challenged by various biotic and abiotic stresses. Recent studies revealed that overexpression of several HSF genes could enhance plants tolerance to multiple stresses (Bi et al., 2020; Poonia et al., 2020), suggesting HSF genes have significant potential for molecular breeding of Triticeae species. Genome-wide analysis of HSF genes has been conducted in wheat and barley, which provides primary resources for mining and utilizing functional HSF genes in the two species (Duan et al., 2019; Zhou et al., 2019; Mishra et al., 2020; Ye et al., 2020). However, the HSF genes composition in rye has not been investigated yet. In this study, we performed genome-wide identification and evolutionary analysis of HSF genes in a recently published rye genome (Li et al., 2021), and performed comparative analysis of HSF genes in Triticeae species.

## Materials and Methods

### Identification and Classification of HSF Family Genes

The genome sequences of *S. cereale*, *H. vulgare*, *T. aestivum* (three subgenomes), *O. sativa* and *A. thaliana* were downloaded from public databases (Table S1). HSF genes were identified as described using a method by Shao et al. (2014) with some modifications. Briefly, the annotated proteins in each genome were screened for the HSF domain (Pfam accession: PF00447) by using the hmmsearch program implemented in the hmmer3.0 software (Johnson et al., 2010). The amino acid sequences of obtained HSF genes were then used to run a genome-wide BLASTp analysis for each genome. All hits were further analyzed using the hmmscan program in hmmer3.0 against the local Pfam-A database to confirm a detectable HSF domain in each sequence, with a e-value setting as 0.0001. All obtained HSF candidates were validated by subjecting to the Heatster database (Fan et al., 2021). Only genes simultaneously encoding DBD, HR-A and HR-B were recognized as true HSF genes, which were classified into the A, B, and C classes by the Heatster database.

### Gene Structural Analyses and Domain Composition Analysis

The gene structure analysis for identified HSFs was constructed using the Gene Structure Display Server (GSDS) (http://gsds.cbi.pku.edu.cn/) (Suyama et al., 2006), while domain composition was presented by using the Tbtools (Chen et al., 2020).

### Sequence Alignment and Phylogenetic Analysis

Amino acid sequences of the HSF domain were aligned using ClustalW program (Edgar, 2004) that is integrated in MEGA 7.0 (Kumar et al., 2016) with default options, and then was manually corrected. ModelFinder was used to estimate the best-fit model of nucleotide substitution (Kalyaanamoorthy et al., 2017). Phylogenetic analyses were performed using the IQ-TREE with the maximum likelihood algorithm (Nguyen et al., 2015). Branch support values were calculated using the SH-aLRT (Anisimova et al., 2011) and the UFBoot2 (Minh et al., 2013) methods with 1,000 bootstrap replicates.

### Synteny Analyses

Inter-species synteny analysis of the HSF genes from *S. cereale*, *T. aestivum* and *H. vulgare* were performed by using the MCScanX program that is integrated in the TBtools (Wang et al., 2012; Chen et al., 2020). Syntenic relationships were then drawn using Tbtools (Chen et al., 2020).

### Gene Expression Analysis

RNA-seq raw reads of rye in different tissues and under different stresses were downloaded from the SRA database (Accesion: SRX9567472). The adaptors were removed using Trim_Galore (https://github.com/FelixKrueger/TrimGalore). The resulted clean reads were mapped to the rye reference genome using Hisat2 (Kim et al., 2019). Quantitation of gene expression was performed using feature Counts (Liao et al., 2014). The resulted read counts of each gene were normalized to FPKM.

## Results

### *Secale cereale* Genome Contains 31 HSF Genes That Are Unevenly Distributed on the Seven Chromosomes

**FIGURE 1:**
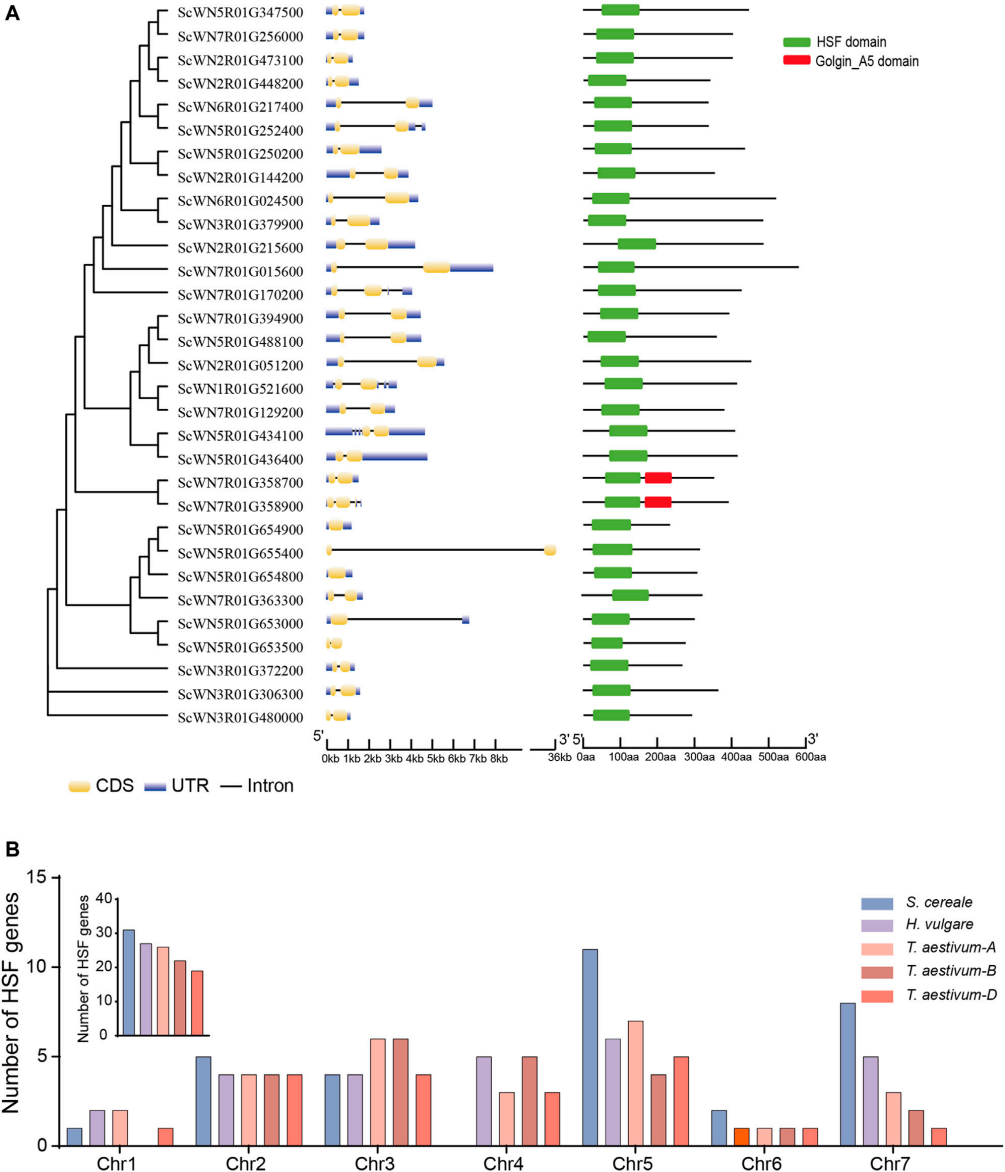
Gene structure, domain composition and chromosome distribution of *S. cereale* HSF genes. **(A)**. HSF genes in the *S. cereale* genome were ordered by the phylogeny. Gene structure and domain composition for each gene were shown following the gene name. **(B)**. The number of HSF genes on each chromosome in *S. cereale* were shown and compared to those of *T. aestivum* and *H. vulgare*.

A total of 31 HSF family members were identified from the *S. cereale* genome by searching the annotated proteome of Weining rye (Figure 1 and Supplementary Table S1). Gene structure analysis revealed that the annotated transcripts for 28 of the 31 HSF genes have both 5′ and 3′ untranslated regions (UTRs), whereas the transcript of one gene (ScWN3R01G480000) only has 3’ UTR and transcripts of two genes (ScWN5R01G655400 and ScWN5R01G653500) do not have annotated UTR region, suggesting an overall high quality of the annotation. The annotated transcripts showed a high diversity of exon-intron composition among different HSF members, with 1–4 introns were found from the transcripts of the 29 genes, and 2 genes not having annotated introns. Among them, 24 HSF genes have only 1 intron, and introns for 23 of them are located at the coding sequences (CDS) of the transcripts. In contrast, 5 transcripts have 2 to 4 annotated introns. The large diversity of exon-intron composition in *S. cereale* may serve as primary resources for generating potential mRNA alternative splicing, which has been reported for several HSF genes in other plants (Ling et al., 2021). The amino acid numbers of the translated proteins from annotated CDSs of HSF genes range from 678 to 1,560 (Supplementary Table S2), suggesting potential fusion of additional domains by some HSF proteins. However, domain structure analysis showed that only two HSF proteins have an additional domain, namely Golgin_A5, at the C-terminal (Figure 1A), indicating a functional innovation of the two HSFs.

Chromosomal distribution analysis revealed that the 31 HSF genes are unevenly distributed on seven chromosomes of *S. cereale*. Chromosome 5 contains the largest number of HSF genes (11 genes), whereas no HSF genes were identified on chromosome 4 (Figure S1). The chromosome 1, 2, 3, 6, 7 have 1, 5, 4, 2, and 8 HSF genes, respectively (Supplementary Figure S1). Since chromosome introgression has been frequently used for Triticeae crops (Li et al., 2021), we compared the chromosomal distribution pattern of HSF genes among *S. cereale*, *H. vulgare*, and *T. aestivum*. The results showed that the overall HSF gene number in *S. cereale* (R genome) is great than that in the *H. vulgare* (H genome) and the subgenome A, B and D of wheat (Figure 1B). In accordance with the high HSF gene number in *S. cereale*, the numbers of HSF genes on the chromosomes 2, 5, 6, and 7 of *S. cereale* each ranks the first among the five genomes, respectively. However, in contrast to the lack of HSF genes on chromosome 4 in *S. cereale*, the *H. vulgare* genome and the subgenome A, B and D of wheat each has 5, 3, 5, and 3 HSF genes, respectively (Figure 1B). Considering the three species were only diverged from the common ancestor within twenty million years, it is possible that the HSF genes on the chromosome 4 of *S. cereale* have translocated to other chromosomes or underwent gene loss.

### Classification of the HSF Genes in *S. cereale* and Four Other Angiosperms Reveals Species-specific HSF Composition

**FIGURE 2:**
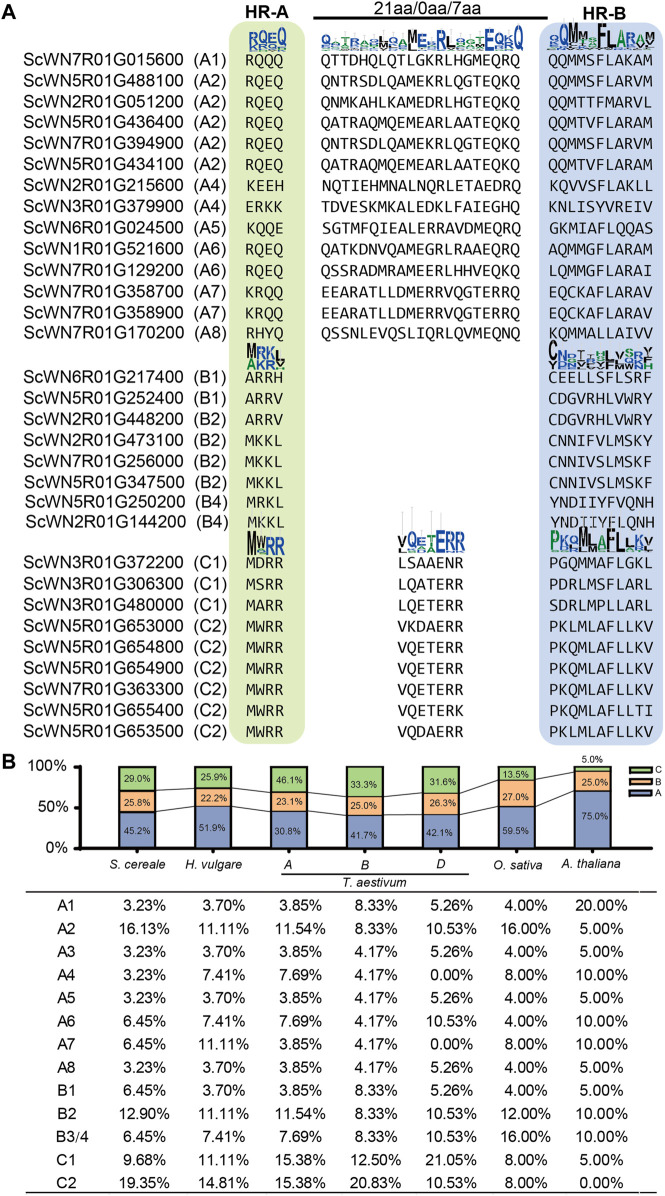
Class and subclass division of *S. cereale* HSF genes. **(A)**. Classification of HSF genes in *S. cereale* based on protein sequence characteristics. **(B)**. Proportion of different classes and subclasses of HSF genes in *S. cereale*, *H. vulgare*, *T. aestivum* (three subgenomes), *O. sativa* and *A. thaliana* respectively.

Plant HSFs have been classified into three classes, HSFA, B, and C, based on the linker length of the DBD and HR-A/B regions and the inserted amino acid residues number into the HR-A/B regions (Scharf et al., 2012). According to this criterion, 14 *S. cereale* HSF genes were assigned to class A, while 8 and 9 genes were assigned to class B and C, respectively (Figure 2A; Supplementary Table S3). The characteristic feature of different insertion size between HR-A/B regions could be clearly observed in the alignment, with 21, and 7 amino acid residues detected in class A and C HSFs, respectively (Figure 2A). No amino acid was detected between the HR-A/B regions for the sequences in class B. The boundary separating HR-A/B regions at the end of HR-A is conserved within each class but differs among the three classes. For example, a conserved motif of ‘RQEQ’ is readily detected at the end of HR-A region in most class A HSFs, whereas nearly all class C HSFs have a ‘MWRR’ motif. In comparison, the boundary of HR-B is less conserved in each class.

The proportion of HSF genes in each class only varied slightly among *S. cereale* (A: 29.0%; B: 25.8%; C: 45.2), *H. vulgare* (A: 25.9%; B: 22.2%; C: 51.9%) and the subgenome B (A: 33.3%; B: 25%; C: 41.7%) and D (A: 31.6%; B: 26.3%; C: 42.1%) of *T. aestivum*. In contrast, the wheat A subgenome showed an elevated proportion of class A (46.1%) HSFs and a decreased proportion of class C (30.8%) HSFs. The HSF genes from *O. sativa* and *A. thaliana* were also identified and their class compositions were compared with those in the three Triticeae species. The results showed that *O. sativa* has a more expanded class A (59.5%) and more contracted class C (13.5%) than that in the wheat subgenome A. The *A. thaliana* genome has the highest ratio of HSFA members (75.0%) and the lowest ratio of HSFC members (5%) among all investigated genomes. This result is consistent with results from previous studies that the class C HSFs have undergone expansion in monocot species (Guo et al., 2016). The proportion of HSFB members is stable among all investigated genomes, ranging from 22.2 to 27.0%. The subclass composition of HSF family also varies cross-species (Figure 2B). For example, subclass C2 occupied the highest proportion of HSFs in *S. cereale*, *H. vulgare* and the wheat subgenome A and B, whereas subclass C1, A2 and A1 occupied the highest proportion of HSFs in the wheat subgenome D, *O. sativa* and *A. thaliana*, respectively.

### Phylogenetic Analysis Reveals Dynamic Loss and Gain of HSF Genes Among Triticeae Species

**FIGURE 3:**
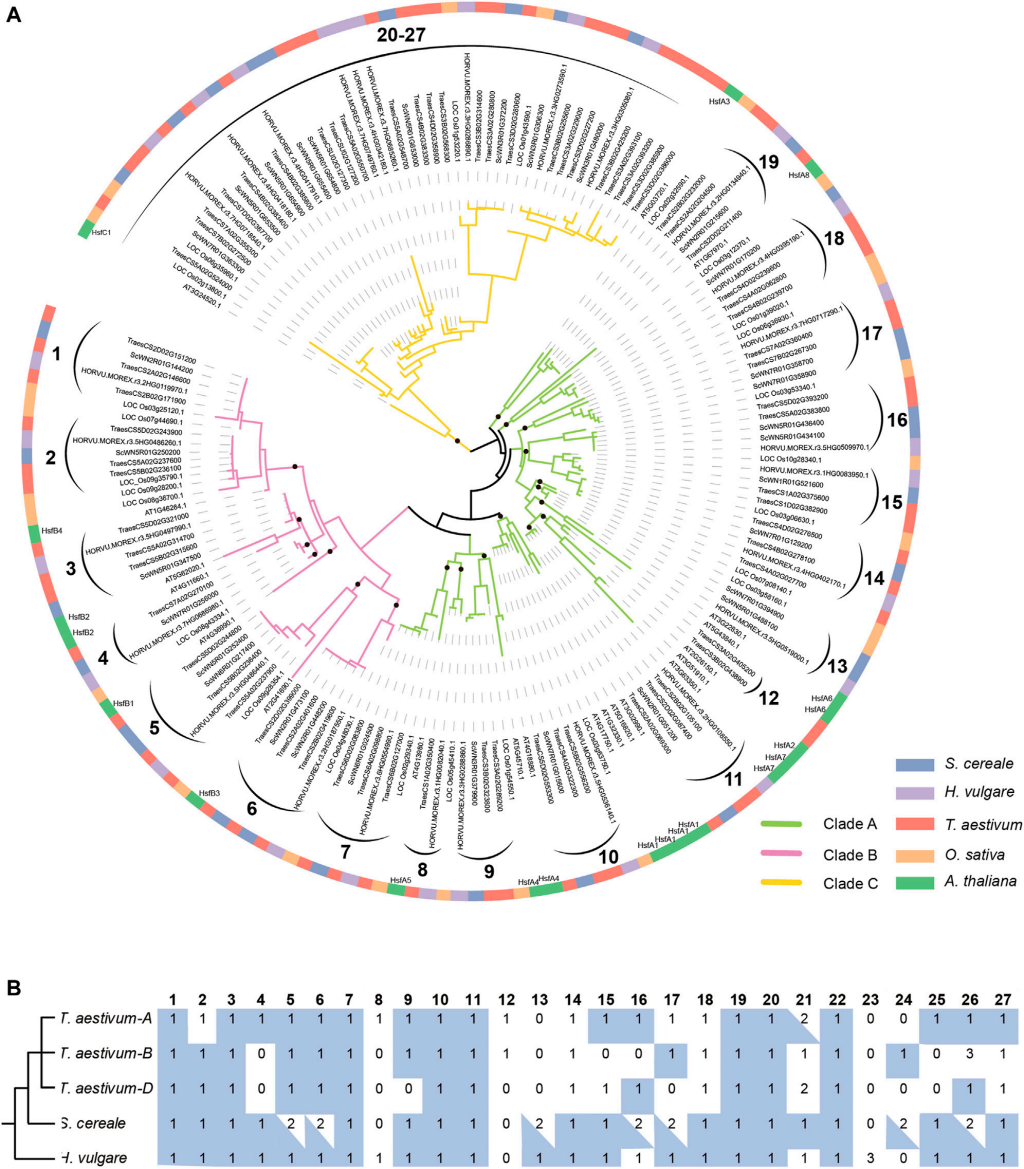
Phylogenetic analysis of HSF genes from *S. cereale, H. vulgare, T. aestivum, O. sativa* and *A. thaliana*. **(A)**. A phylogeny of HSF genes from *S. cereale*, *H. vulgare*, *T. aestivum* (three subgenomes), *O. sativa* and *A. thaliana* based on the amino acid sequences of the HSF domain. Black brackets indicate a group of orthologous HSFs in Triticeae. The identification of class C Triticeae HSF orthologous group were based on an additional phylogeny provided in Supplementary Figure S2, which is constructed by using the full-length protein sequences encoded by HSFC genes. Black dots on the basal of several lineages indicate ancestral HSF lineages of monocots and dicots. **(B)**. The number of HSF genes from different species in each Triticeae HSF orthologous group. A blue rectangle background indicates the HSF gene and HSF genes from at least one other species are in collinear blocks. A blue triangle indicates one of the HSFs in this orthologous group has a collinear relationship with HSF genes from at least one other species.

To clarify the evolutionary relationship of HSF genes among *S. cereale*, *H. vulgare*, and *T. aestivum*, and trace the evolutionary trajectory during species-speciation of Triticeae, a phylogenetic analysis was performed for HSF genes from the three Triticeae species with those from *O. sativa* and *A. thaliana* (Figure 3A). The result showed that members of HSFB and HSFC form two separate monophyletic clades, containing 43 and 47 genes respectively, whereas members of HSFA form a paraphyletic group containing 81 genes. The topology of the HSF phylogeny is also highly consistent with a previous study which showed that HSFC may be diverged from HSFA in a rooted plant HSF phylogeny (Wang et al., 2018). This HSF phylogeny also supports the classification result based on characteristic features of the protein sequences. Tracing the evolutionary history of different HSF classes revealed that the monocot and dicot HSFA genes are inherited from at least 13 ancestral lineages, while the HSFB genes are inherited from 7 ancestral lineages that are presented in the common ancestor of monocot and dicot species (Figure 3A). Interestingly, there is only 1 *A. thaliana* (At3G24520) gene presented in the HSFC clade which contains 46 monocot sequences (Figure 3A), including 9, 9, 24 and 4 genes from *S. cereale*, *H. vulgare*, *T. aestivum* and *O. sativa*, suggesting the ancestral HSFC lineage that presented in the common ancestor of monocot and dicot has experienced drastic expansion in monocot species.

Further analysis of the phylogeny revealed that HSF genes from the *S. cereale*, *H. vulgare* and *T. aestivum* form 27 independent groups, suggesting that the 21 ancestral HSF genes in the common ancestor of monocot and dicot further diverged into at least 27 Triticeae HSF lineages before the separation of the three Triticeae species (Figure 3A). Interspecific synteny analysis showed that 24 genes of the 27 Triticeae HSF lineages could be detected at syntenic chromosomal blocks from at least two species, providing additional evidence to support the orthologous relationship of genes in each lineage. Interestingly, several interspecies syntenic blocks were detected among chromosome 7 of *S. cereale*, chromosome 4 or 5 of *H. vulgare* and/or the subgenome A, B of wheat (Supplementary Figure S3; Supplementary Table S4). The result suggests that chromosome rearrangement may have occurred in the *S. cereale* genome, causing HSF genes escaped from its chromosome 4.

Different extent of gene duplication and gene loss could be traced from *S. cereale*, *H. vulgare*, and the three subgenomes of *T. aestivum*. Among them, *S. cereale* has the most duplicated genes for the 27 Triticeae HSF lineages. Seven of the Triticeae HSF lineages have duplicated in the *S. cereale* genome, including 3, 2 and 2 lineages in the HSF A, B and C classes, respectively (Figures 3A,B; Supplementary Figure S2). The duplicated gene pairs are presented at adjacent, distant region of the same chromosome or at different chromosomes, suggesting tandem, dispersed, small-scale segmental duplications and ectopic duplications have been evolved in generating new HSF copies in the genome. In contrast, *H. vulgare* and the three subgenomes of *T. aestivum* each has one duplicated Triticeae HSF lineage. Loss of Triticeae HSF lineages was also detected in the three species. The *H. vulgare* genome lost 2 Triticeae HSF lineages (lineage 12 and 24), while the *S. cereale* and the subgenome A, B and D of wheat lost 3, 3, 7 and 9 Triticeae HSF lineages, respectively.

### A Majority of *S. cereale* HSF Genes Show Tissue-Specific or Developmental Stage-Dependent Expression and can Response to Multiple Stresses

**FIGURE 4:**
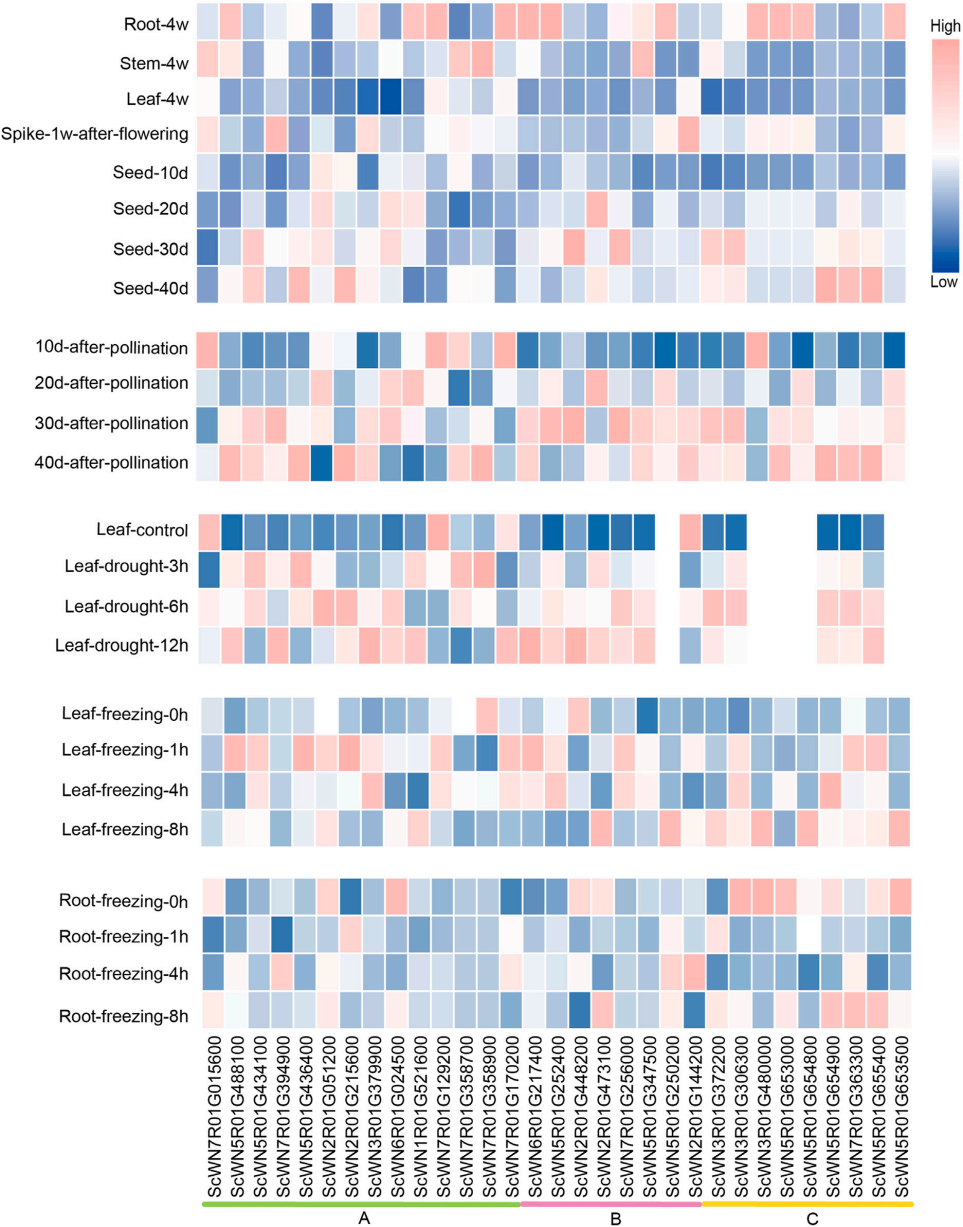
Expression analysis of HSF genes in *S. cereale* across different tissues, development stages and different stress treatments.

To explore the potential involvement of HSF members in development and resistance to environmental stresses, we analyzed their expression patterns using the public data (Li et al., 2021). An obvious tissue-specific or developmental stage-specific high expression was observed for nearly all HSF members (Figure 4). Furthermore, the expressions of most HSF genes are higher in root than in leaf and stem, except four genes, including 3 from the class A and 1 from the class B, which have the highest expression in stem among the three tissues (Figure 4). During *S. cereal* development after flowering, four genes show the highest expression in spike 1 week after flowering, whereas five genes have the highest expression in 40-days seed. The expression levels of most HSFs gradually increased during 10-days to 40-days after pollination.

We also analyzed the expressions of HSFs in leaves and roots under drought and freezing conditions. The results show that most HSF genes were induced by drought in leaf at least at one time point compared to the control, except ScWN7R01G015600, ScWN7R01G129200 and ScWN2R01G144200, which were down-expressed at the drought condition. A similar pattern was also observed for a large number of HSF genes in freezing leaves, except ScWN7R01G358900 and ScWN2R01G448200, the expressions of which were repressed by freezing treatment. In contrast to the pattern observed in leaves, only a few HSFs showed induced expression upon freezing treatment in root, whereas a considerable number of HSFs genes were down-expressed (Figure 4). The result suggests that leaf and root may adapt different combinations of HSF genes to respond to the freezing stress. We also observed expression divergence of a newly duplicated gene with its parental gene. The ScWN7R01G394900, which is duplicated by ectopic duplication from the ScWN5R01G488100, shows the highest expression in spike, whereas ScWN5R01G488100 only highly expressed in root but not in other all detected tissues. Similar pattern of expression divergence could also be detected in stress treatment. ScWN7R01G394900 was induced in root upon freezing treatment, whereas ScWN5R01G488100 showed no obvious expression alteration. The results suggest functional divergence may have occurred in the gene pairs.

## Discussion

Understanding the molecular mechanisms of how plants respond to abiotic and biotic stresses is important for improving plant tolerance to stresses and crop productivity. HSF family proteins can modulate the expression of genes in responding to heat, cold, salt stresses and pathogens (Andrasi et al., 2021). The present study identified 31 HSF genes from the recently released *S. cereale* genome and traced the dynamic evolution of the HSF genes in three Triticeae species. *S. cereale* is an important cereal crop, which has high tolerance to many biotic and abiotic stresses (Li et al., 2021). The identification of HSF genes from *S. cereale* in this study provides a primary resource for mining functional genes that will facilitate the molecular breeding of *S. cereale*. Moreover, *S. cereal* plays an important role in the improvement of wheat breeding (Merker, 1984). It has great potential to expand the genetic variability of *T. aestivum*. Actually, genes with different functions have been transformed from *S. cereal* to *T. aestivum* to improve the growth or resistance to biotic and abiotic stress (Szakacs et al., 2020). The *S. cereale* HSF genes should also be served as a potential resource for wheat and other crops’ breeding.

Different numbers of HSF genes have been identified from model and crop plants (Wang et al., 2018), however the evolutionary history of HSF genes in a specific plant lineage has rarely been investigated. By incorporating HSF genes from *O. sativa* and *A. thaliana* for the phylogenetic analysis, we found that the HSF gene family had undergone extensive expansion prior to the divergence of monocot and dicot, with at least 21 ancestral lineages being recovered. This number exceeded the quantity of the currently defined HSF subclass (Berz et al., 2019), suggesting an updated subclass definition by including HSF genes from more plant genomes is needed to help functional distinguish anciently diverged lineages. The ancestral HSF lineages further expanded before the radiation of Triticeae, with at least 27 ancestral Triticeae HSF lineages could be traced to the common ancestor of the three Triticeae species. These genes were differentially inherited by *S. cereale*, *H. vulgare*, and *T. aestivum*. *S. cereale* lost three of the ancestral Triticeae HSF lineages that presented in the common ancestor the three Triticeae species, while *H. vulgare* and *T. aestivum* both lost two. However, the *S. cereale* genome has more HSF genes than the *H. vulgare* genome and the three *T. aestivum* subgenomes, because of more specie-specific gene duplications and fewer gene loss events have been occurred after it separated with the other two species. A recent study of wheat revealed that both A subgenome and D subgenome have more HSF genes than their wild ancestors (*T. urartu*, 15 genes and *A. tauschii*, 16 genes) and concluded that the number of HSF increased in transition from diploidy to hexaploidy (Zhou et al., 2019). However, our data does not support this notion, because only one gene has gained by the wheat A genome after its separation from rye and barley, whereas three gene lost occurred. Similarly, only one gene has gained by the wheat D genome after its separation from rye and barley, whereas 9 gene lost occurred. Therefore, the fewer HSF genes in the genome of *T. urartu* and *A. tauschii* than those in wheat A and D subgenomes is more likely due to the increased gene loss in the two genomes.

Gene duplication provides raw resource for gene function innovation (Guo et al., 2019). As shown by the expression data, HSF genes from different ancestral lineages show diverse expression patterns across different tissues, development stages and stress treatments. This is consistent with the previous studies in wheat, suggesting multiple functions of HSF genes (Ye et al., 2020). Recent gene duplications also contribute to plant adaptive evolution (Zhang and Long, 2014). While few HSF gene duplications were detected in the barley genome, the wheat genome obviously benefitted from harboring three subgenomes to have a neatly tripled HSF number. The *S. cereale* genome adopted a different strategy to amplify its HSF content, with six gene duplications generated by different mechanisms detected. The newly birthed genes provide opportunities for functional innovation of HSF genes in *S. cereale*. To support our speculation, we found that a pair of genes from one duplication showed inconsistent expression patterns, suggesting functional innovation may have occurred in the recently duplicated *S. cereale* HSF genes.

## Conclusion

In summary, this study presents a complete profile of HSF genes in *S. cereale*, which is composed by 31 genes from three classes. Chromosomal reorganization may have contributed to the HSF escape from chromosome 4 in *S. cereale*. Phylogenetic and syntenic analysis supported that at least 27 ancestral HSF lineages were presented in the common ancestor of *S. cereale*, *H. vulgare*, and *T. aestivum*. *S. cereale* experienced the most HSF gene duplications among the Triticeae A, B, D, R and H genomes. Expression analysis revealed the potential involvement of HSF genes in growth, development and response to abiotic stress of *S. cereale*, and indicated the functional innovation of recently duplicated HSF genes. The results provide new insights into the evolution of HSF genes in Triticeae and may serve as a resource for Triticeae molecular breeding.

## Data Availability

The original contributions presented in the study are included in the article/Supplementary Material, further inquiries can be directed to the corresponding authors.
