# Supplementary figures and images for: Comparative Analysis of HSF Genes From Secale cereale and its Triticeae Relatives Reveal Ancient and Recent Gene Expansions
Source: Front Genet. 2021 Nov 23;12:801218. doi: 10.3389/fgene.2021.801218 (PMC8650501; doi:10.3389/fgene.2021.801218)

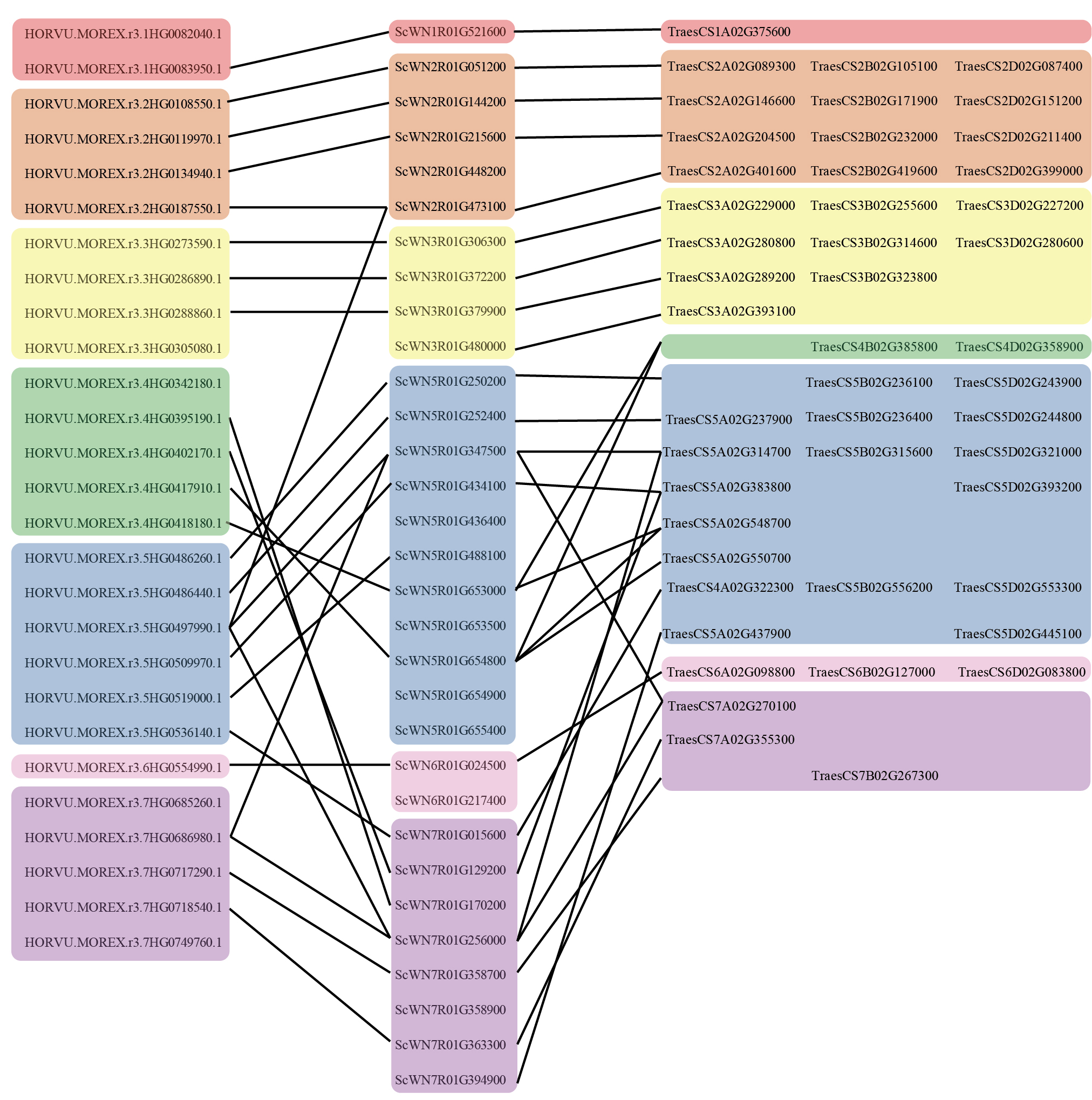

Supplement: Supplementary file 2 [file Image3.JPEG]

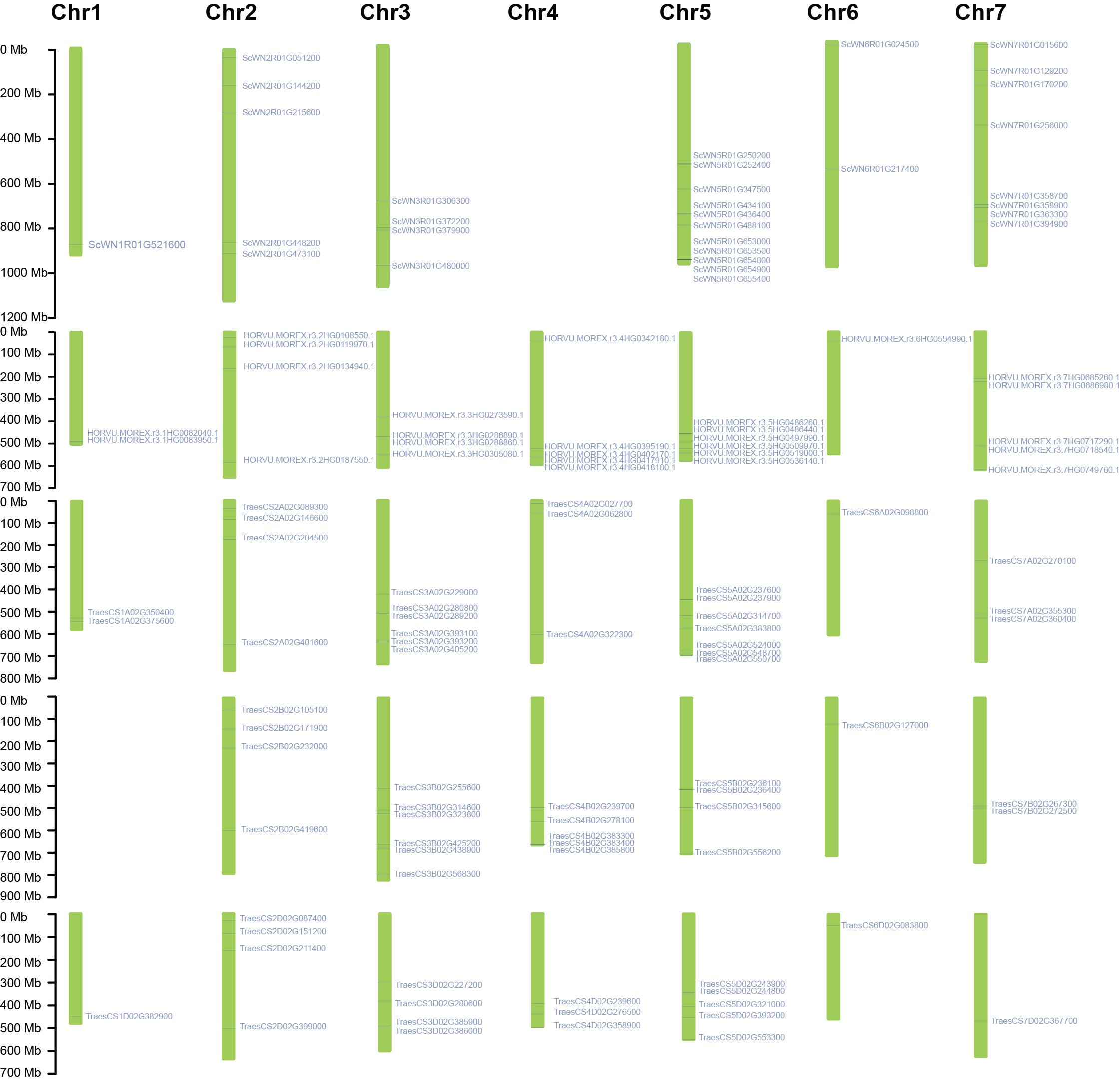

Supplement: Supplementary file 4 [file Image1.JPEG]

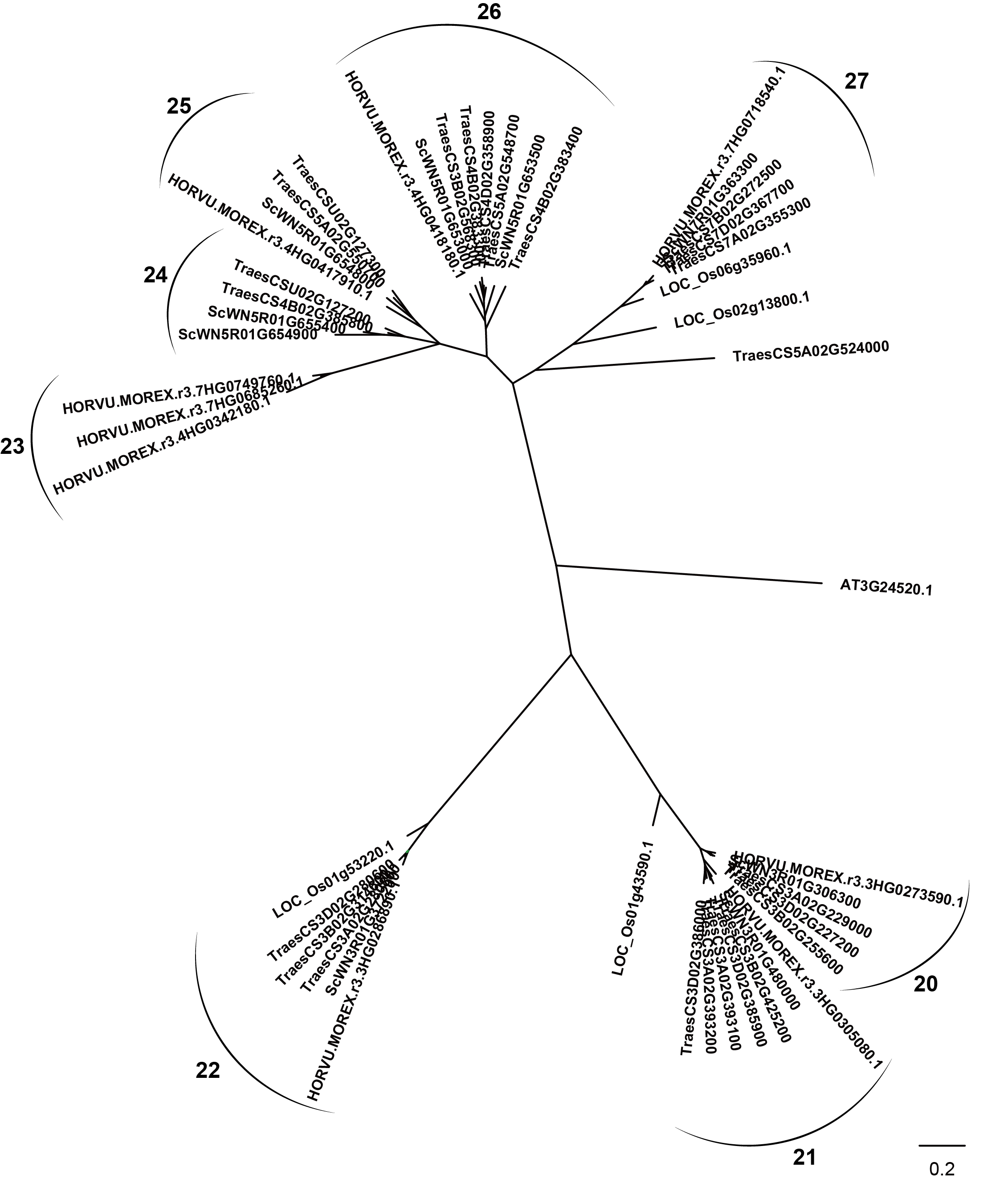

Supplement: Supplementary file 5 [file Image2.JPEG]
